# Glycomic Approaches for the Discovery of Targets in Gastrointestinal Cancer

**DOI:** 10.3389/fonc.2016.00055

**Published:** 2016-03-09

**Authors:** Stefan Mereiter, Meritxell Balmaña, Joana Gomes, Ana Magalhães, Celso A. Reis

**Affiliations:** ^1^Instituto de Investigação e Inovação em Saúde (I3S), University of Porto, Porto, Portugal; ^2^Institute of Molecular Pathology and Immunology of the University of Porto (IPATIMUP), Porto, Portugal; ^3^Institute of Biomedical Sciences of Abel Salazar (ICBAS), University of Porto, Porto, Portugal; ^4^Biochemistry and Molecular Biology Unit, Department of Biology, University of Girona, Girona, Spain; ^5^Medical Faculty, University of Porto, Porto, Portugal

**Keywords:** gastric cancer, colorectal cancer, pancreatic cancer, glycomics, glycan biomarkers, microarray, proximity ligation assay, imaging mass spectrometry

## Abstract

Gastrointestinal (GI) cancer is the most common group of malignancies and many of its types are among the most deadly. Various glycoconjugates have been used in clinical practice as serum biomarker for several GI tumors, however, with limited diagnose application. Despite the good accessibility by endoscopy of many GI organs, the lack of reliable serum biomarkers often leads to late diagnosis of malignancy and consequently low 5-year survival rates. Recent advances in analytical techniques have provided novel glycoproteomic and glycomic data and generated functional information and putative biomarker targets in oncology. Glycosylation alterations have been demonstrated in a series of glycoconjugates (glycoproteins, proteoglycans, and glycosphingolipids) that are involved in cancer cell adhesion, signaling, invasion, and metastasis formation. In this review, we present an overview on the major glycosylation alterations in GI cancer and the current serological biomarkers used in the clinical oncology setting. We further describe recent glycomic studies in GI cancer, namely gastric, colorectal, and pancreatic cancer. Moreover, we discuss the role of glycosylation as a modulator of the function of several key players in cancer cell biology. Finally, we address several state-of-the-art techniques currently applied in this field, such as glycomic and glycoproteomic analyses, the application of glycoengineered cell line models, microarray and proximity ligation assay, and imaging mass spectrometry, and provide an outlook to future perspectives and clinical applications.

## Glycobiology in Cancer

The cells’ glycocalix constitutes an important interface with the extracellular milieu and plays critical roles in physiological and pathological conditions. This glycan-rich coating of the cells’ plasma membrane is composed by different classes of glycoconjugates, including glycoproteins, glycolipids, and proteoglycans, which participate in key regulatory events for cellular and organ homeostasis. Alterations in glycosylation can interfere with normal molecular functions such as cell–cell recognition, communication, and adhesion, leading to acquisition of malignant features. Moreover, the shedding of aberrant glycoconjugates, uniquely expressed by tumor cells, into circulation provides one valuable source of biomarkers for cancer diagnosis and prognosis (1).

Substantial advances in the frontiers of cancer glycobiology have been possible in the recent past due to the combination of novel tumor cell biology concepts with cutting-edge glycomic technologies. Specific glycosylation alterations have been identified in tumors and some of the molecular pathways underlying these modifications have been disclosed (2). In addition, aberrant glycoforms have been demonstrated to be molecularly associated with more aggressive cancer cell and tumor features, including increased migration, invasion, and metastization potential, providing novel targets for therapeutic intervention (3–5).

This review describes the recent progress in gastric cancer, colorectal cancer (CRC), and pancreatic ductal adenocarcinoma (PDAC) glycobiology and discusses the clinical value of aberrant glycosylation as a source of screening biomarkers and therapeutic targets. A comprehensive overview of the advances in glycomic and glycoproteomic tools is also provided and their possible applications for tumor glycan-profiling and discovery of novel targets for improving gastrointestinal (GI) tumors’ clinical management are discussed.

## Glycosylation Alteration in Gastrointestinal Cancers

**Figure 1:**
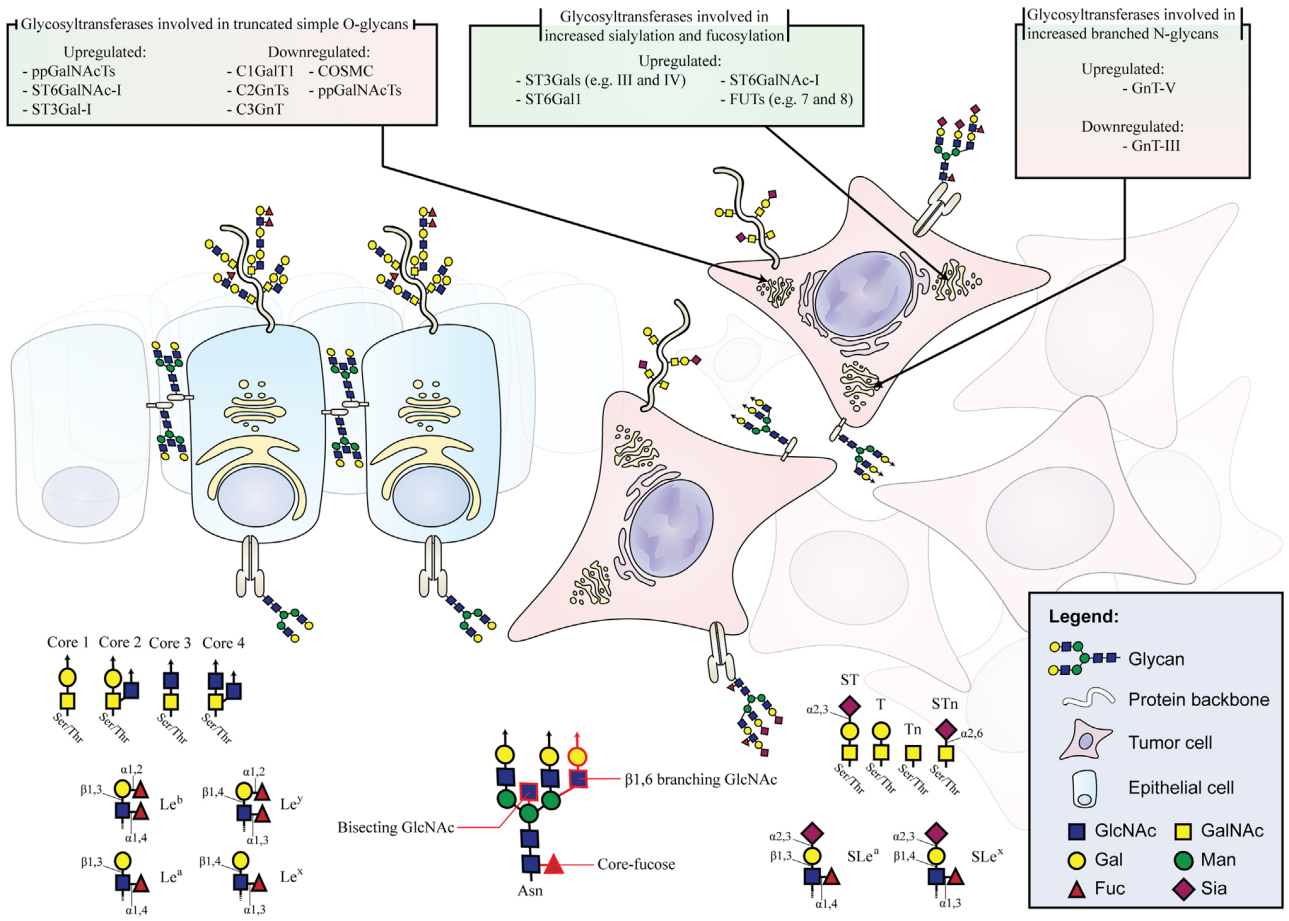
**Schematic depiction of glycan alterations during malignant transformation**. Healthy epithelium displaying cell polarity, organized glycoprotein localization, and normal glycosylation pattern. Malignant cells with misslocalized glycoproteins and altered expression of genes involved in glycosylation pathways leading to the aberrant expression of glycan moieties. The glycosyltransferases involved in glycan alterations during malignant transformation are listed.

Despite the large amount of glycan epitopes that can be found in the human GI tract and the complex and manifold alterations of the glycosylation machinery during the process of carcinogenesis and cancer progression, the current knowledge makes it possible to group the most common glycan alterations. Expression of truncated simple *O*-glycans, changes in *N*-glycan branching, and increase in sialylation and fucosylation are three major *N*- and *O*-glycosylation events involved in GI cancer that will be described in detail (Figure 1). Furthermore, we give an overview on other common glycosylation alterations in cancer, such as changes in *O*-GlcNAcylation, modified glycosphingolipids, and glycosaminoglycans (GAGs) and proteoglycans.

### Truncated Simple *O*-Glycans

One common feature observed in GI tumors is the overexpression and exposure of short, truncated *O*-glycans. Mucin-type *O*-glycans are found on most transmembrane and secreted proteins. A single *O-*glycan oligosaccharide chain can present more than 20 monosaccharide constituents (6). In malignancy, *O*-glycans are often shortened resulting in an increase of the monosaccharide Tn antigen (GalNAcα1-Ser/Thr), the disaccharide T antigen (also known as Thomsen–Friedenreich antigen or core 1 structure, Galβ1-3GalNAcα1-Ser/Thr) and their sialylated forms, STn (Neu5Acα2-6GalNAcα1-Ser/Thr), and ST (Neu5Acα2-3Galβ1-3GalNAcα1-Ser/Thr), respectively (Figure 1) (7, 8).

Polypeptide GalNAc transferases (ppGalNAcTs), which are the initiating enzymes of the mucin-type *O*-glycosylation (9, 10), show often altered expression in cancer (11–13). A total of 20 different ppGalNAcTs are known in human and their expression profile and subcellular localization determine *O*-glycosylation sites and densities (9, 14). In CRC, for example, the ppGalNAc-T3 is associated with tumor differentiation, disease aggressiveness, and prognosis (12). In gastric cancer, the expression of ppGalNAc-T6 is associated with venous invasion and the downregulation of ppGalNAc-T2 increases the cancer cell proliferation, adhesion, and invasion (11, 15).

In addition, enzymes competing for the same substrate can also induce expression of truncated glycans and exposure of protein epitopes that would be hidden otherwise. For instance, the relative enzymatic activities of C2GnT (*N*-acetylglucosaminyltransferase) and ST3Gal-I (sialyltransferase), two glycosyltransferases that compete for the same substrate, have been shown to determine the *O*-glycan structure in cancer cells (16).

STn is expressed in most GI carcinomas correlating with decreased cancer cell adhesion, increased cancer cell invasion, and poor prognosis of the patients (17–23). The terminal STn epitope is synthesized by the sialylation of Tn by the ST6GalNAc-I sialyltransferase (17, 18). In cancer, the formation of STn may occur due to ST6GalNAc-I upregulation, early sialylation caused by glycosyltransferase misslocalization in the secretory pathway, or the impairment of the elongation of the Tn antigen (14, 17, 18, 24).

In gastric cancer, expression of STn is a common feature associated with more malignant phenotypes. The overexpression of ST6GalNAc-I has been shown to induce migration and invasion in gastric carcinoma cells *in vitro* (20).

In this regard, another gene that can underlie the synthesis of truncated *O*-glycans is *COSMC*, which encodes for a C1GalT1 dedicated chaperone (25). The galactosyltransferase C1GalT1 is responsible for the elongation of the Tn antigen to form the core 1 structure also known as the T antigen. The absence of a functional *COSMC* entails the dysfunction of C1GalT1. In PDAC, it has been shown that hypermethylation of *COSMC*, and not somatic mutations, is the prevalent cause of truncated *O*-glycans (23). In addition, the downregulation of C1GalT1 in combination with the upregulation of ST6GalNAc-I has been associated with increased STn expression in CRC cell lines and epithelial cells derived from resected CRC tumor tissue (26). In contrary, the overexpression of C1GalT1 is associated with invasion, metastization, and poor survival in CRC. In C1GalT1 overexpressing CRC cells, the knockdown of C1GalT1 suppresses the malignant phenotype *in vitro* and *in vivo* (27). Increased levels of C2GnT, a glycosyltransferase responsible for the biosynthesis of core 2 structures, are also frequent in CRC (28). This enzyme has also a critical role in the biosynthesis of terminal sialylated Lewis antigens on *O*-glycans that will be further discussed in Section “Increased Sialylation and Fucosylation.”

Normal pancreas does not express the Tn antigen and its corresponding sialylated epitope STn (21). The Tn antigen is detected in 75–90% of PDACs and up to 67% in precursor lesions (24). The appearance of the STn in mucins, on the other hand, is a late event in PDAC disease progression (29). These truncated *O*-glycans are associated with cancer cell growth and tumor invasion in PDAC (23, 24). The situation is slightly different in CRC, where the overexpression of T antigen is associated with early events in cancer progression and both Tn and STn antigens are frequently overexpressed in advanced and poorly differentiated adenocarcinomas and also in mucinous carcinomas. Therefore, these antigens are considered useful markers for poor outcome (22).

Besides cores 1 and 2, *O*-glycans with cores 3 and 4 are also often expressed in normal GI epithelia, especially in colon, but are significantly decreased in malignancy due to downregulation of the core 3 and 4-synthetase (8, 30–32).

The *de novo* expression of truncated *O*-glycans in GI cancers is of avail in the search of specific cancer biomarkers. It leads to the expression of unique glycopeptide structures and, because of the small steric size of these truncated *O*-glycan moieties, to the exposure of protein regions that would otherwise be masked, and, therefore, not detected by specific antibodies (29, 33).

### Branched *N*-Glycans

The biosynthesis and maturation of *N*-glycan structures is defined by a complex interplay of numerous glycosidases and glycosyltransferases in the endoplasmic reticulum and Golgi. Among *N*-glycan types, the complex *N*-glycans display the largest structural diversity. Two structural features of complex *N*-glycans are the β1,6-branching, catalyzed by the glycosyltransferase GnT-V, and the bisecting-GlcNAc, added by the glycosyltransferase GnT-III. GnT-V is known to be upregulated in gastric carcinoma (Figure 1) (34), leading to the increased branching of *N*-glycans and contributing to cancer cell invasion and metastases (35, 36). Analogically, normal colon epithelium presents high levels of bisecting-GlcNAc, due to high expression levels of GnT-III, which is associated with suppression of the tumor progression. However, during cancer progression, these bisecting structures are decreased (37) and it has been described a general increase of β1,6-branched in complex *N*-linked glycans that are also associated with tumor invasion and metastasis (38). Histochemical studies using specific lectins for the detection of β1,6-branched structures showed increased staining concomitant with tumor CRC staging (39), and an association with lymph node metastasis and decreased survival rates in CRC patients (40). GnT-V, the enzyme responsible for the synthesis of β1,6-branched *N*-glycans, is commonly upregulated in CRC correlating to the metastatic potential and consequently considered an important prognosis factor to detect poor CRC patients’ outcome (41). A recent study demonstrated that GnT-V levels modulate CRC stem cells and tumor formation by Wnt signaling (42). Increased extension of β1,6-branched complex *N*-glycans by long polymers of *N*-acetyllactosamine (LacNAc) due to upregulation of β3GnT8 has also been described in CRC cells (43).

Regarding PDAC progression, little has been described about bisecting structures, although the increase in highly branched *N*-glycans is well established. The number of tri- and tetra-antennary glycans is significantly increased in both pancreatic cancer cells and PDAC patients’ serum and correlate with cancer progression (44, 45).

Another mechanism leading to increased branching is the downregulation of GnT-III and the addition of bisecting-GlcNAc. Bisected structures cannot be further modified by GnT-V and, therefore, preclude the formation of branched *N*-glycans under healthy conditions.

The interplay of GnT-III and GnT-V modulates cell adhesion and migration in a gastric cancer context (46, 47). This has been shown to be particularly important for cell–cell and cell–matrix interactions in gastric cancer by altering the functionality of E-cadherin and integrins in malignant transformation. E-cadherin promotes adherence junction formation and, thus, maintains intercellular adhesion. E-cadherin is stabilized by bisected *N*-glycans delaying its endocytosis and turnover (47–49). Furthermore, bisecting-GlcNAc on E-cadherin is gastric tumor suppressive by downregulating signaling pathways involved in cell motility and the EMT process (50–54). Conversely, E-cadherin is dysregulated when glycosylated with branched *N*-glycans by GnT-V in the context of gastric cancer (34, 52, 53). GnT-V is commonly upregulated in gastric carcinomas contributing to cell invasion and metastases (35, 36). The overexpression of GnT-V leads to destabilization of adherence junctions, delocalization of E-cadherin into the cytoplasm, and mesenchymal appearance of the cells with increased metastatic capability (34, 52, 55).

Integrins convey adhesion to extracellular matrix components and are often altered in GI carcinomas. In gastric cancer, the modification of α3β1 integrin with branched *N*-glycans increases cell migration (56). The modification of α3β1 integrin with bisecting-GlcNAc has the opposite effect by inhibiting cell migration (56). Consistently, the overexpression of GnT-III resulted in the inhibition of α5β1 integrin-mediated cell migration and reduced binding to fibronectin due to a specific *N*-glycosylation site on the α5 integrin (57, 58).

### Increased Sialylation and Fucosylation

Sialic acids are the largest and the only intrinsically negatively charged monosaccharides present in human glycosylation. As a terminal event, sialylation caps glycosylation chains usually resulting in exposed locations of the negative charge at the forefront of the oligosaccharides and first encounter point for adjacent glycans, proteins, and cells. Sialylation has, therefore, been shown to play important roles in modulating cellular recognition, cell adhesion, and cell signaling (59). Moreover, cancer cell sialylation patterns define sialic acid-binding lectins (Siglecs) interactions and modulate immune response (60, 61).

An increase in global sialylation, especially in α2,6- and α2,3-linked sialylation, owing to altered glycosyltransferases expression, has been closely associated with cancer and commonly described as one of the main modifications in GI cancers (62, 63). For example, ST6Gal-I, the enzyme that adds α2,6-linked sialic acid to lactosamine chains (Neu5Acα2,6Galβ1,4GlcNAc), is commonly overexpressed in GI cancers correlating with poor prognosis (59, 64, 65). Additionally, α2,3-sialyltransferases, such as ST3Gal-III and ST3Gal-IV, are often upregulated in the course of gastric cancer and PDAC progression leading to a more invasive and metastatic phenotypes of the cancer cells (65–69). Furthermore, sialylation, in particular α2,3 and α2,6-linked, can modulate the ECM adhesion and migration. Specifically, it has been described that while the overexpression of terminal α2,6-linked sialic acid leads to increased ECM adhesion, the overexpression of α2,3-linked terminal sialic acid epitopes in PDAC cancer cell lines results in a more migratory phenotype (70). Similarly, in gastric cancer cells, the overexpression of α2,3-linked terminal sialic acid epitopes causes a more invasive phenotype *in vitro* and *in vivo* (67).

The major α2,3-sialylated antigens associated with cancer are SLe^a^ and SLe^x^ (Figure 1). Although these structures can also be present in non-neoplastic cells, SLe^a^ and SLe^x^ have been demonstrated to be highly expressed in many malignant tissues, including GI tumors, both in glycoproteins and glycosphigolipids (71–74). SLe^x^-increased expression levels are associated with advanced stages and have been correlated with poor survival in GI cancer patients (75–77). SLe^x^ is the well-known ligand for selectins (78). During inflammation, selectins mediate the initial attachment of leukocytes to the endothelium during the process of leukocyte extravasation. In cancer, SLe^x^ interactions with selectins favor metastasis by forming emboli of cancer cells and platelets and promoting their arrest on endothelia (77).

The overexpression of SLe^x^ in a gastric carcinoma cell line transfected with *ST3GAL4* has shown to increase the cells invasive potential both *in vitro* and *in vivo* due to the activation of the oncogenic c-Met receptor tyrosine kinase (67). Moreover, overexpression of *ST3GAL4* has been shown to result in RON receptor tyrosine kinase activation and co-expression of RON and SLe^x^ is observed in gastric tumors (79). This is of particular biological relevance since it has been described that RON activation contributes to tumor progression, angiogenesis, and therapy resistance and correlates with bad prognosis (80–84).

Sialylated Lewis epitopes are potential good markers for prognosis due to their high incidence of recurrence or presence in metastasis and correlation with the tumor stage. For example, a recent work described the increase of the SLe^x^ epitope on ceruloplasmin in PDAC. The increased ceruloplasmin with the SLe^x^ epitope in chronic pancreatitis was lower, suggesting good specificity for pancreatic malignancy (85). Moreover, studies using high-density antibody microarray also detected increased levels of SLe^x^ and SLe^a^ antigens on glycoproteins in serum or plasma of CRC patients (86).

Overexpression of the enzyme β-galactoside α2,6-sialyltransferase I (ST6Gal-I), especially in *N*-glycans and not in *O*-glycans, has been associated with CRC progression, increased invasion, and metastization and consequently poor prognosis in CRC patients (64, 87). Further studies taking into consideration the low levels of ST6Gal-I in healthy individuals and upregulation in CRC patients could lead to the development of new diagnostic and therapeutic targets.

Fucosylation is also an important modification involved in cancer and inflammation (88). The attachment of fucose to *N*-glycans, *O*-glycans, and glycolipids has been reported in cancer tissues, regulating different biological processes, and being also responsible for the increased expression of Lewis antigens (89) (and sialylated-Lewis antigens, as previously described). Different studies link the presence of fucosylated epitopes on specific glycoproteins with cancer. In particular, research performed on some acute phase proteins suggest the suitability of fucosylated epitopes for cancer management. It has been demonstrated that fucosylated alpha-fetoprotein (AFP) is more specific as a hepatocellular carcinoma biomarker than AFP itself. Nowadays fucosylated AFP (AFP-L3) is used for hepatocellular carcinoma risk assessment (90, 91). Acute phase proteins, such as AFP, are proteins synthesized by hepatocytes and have shown clinical value as markers for liver and pancreatic-related diseases. For example, haptoglobin and AGP have revealed an increase in fucosylated epitopes that could help to improve PDAC diagnosis (89, 92).

Increased activity in α1,3 and α1,4 fucosyltransferases (FUTs) was described in CRC patients, resulting in the synthesis of SLe^x^ and SLe^a^ epitopes, respectively (89). Particularly, FUT6 was more recently reported as the major regulator of SLe^x^ biosynthesis in CRC (93). Increased levels of fucosylation in plasma samples of CRC patients compared to normal controls were also described using methods for *N*-glycoproteomics analysis to identify plasma markers (94). In addition, increased levels of α1,2-FUT1 and FUT2, which add fucose to terminal galactose and are essential for the synthesis of Lewis Y and B antigens, were shown in CRC tumors (95). Alterations of FUT expression have also been described in the process of gastric carcinogenesis (96). In particular, the downregulation of FUT3 and FUT5 changes the Lewis antigens expression and reduces the adhesion capacities of gastric cancer cells (97). This is contrary to what is observed in gastric inflamed mucosa, where FUT3 is upregulated (98).

Increased core-fucosylation of *N*-glycans catalyzed by α1,6-FUT8 has been described in CRC patients and is associated with tumor aggressiveness (37, 99). The core-fucosylation of E-cadherin enhances the cellular adhesion of CRC cells (100). However, in gastric cancer, the decrease of core-fucosylation has been demonstrated to be a common event contributing to cancer cell proliferation (101).

### Other Relevant Glycosylation Alterations

In addition to the mucin-type *O*-glycosylation, there are further forms of protein *O*-glycosylation, including the modification of nuclear and cytoplasmic proteins with *O*-linked β-*N*-acetylglucosamine (*O*-GlcNAc). Noteworthy, increased *O*-GlcNAcylation is a general feature of cancer and the modification of proteins with *O-*GlcNAc has been shown to play key regulatory roles in tumor cell signaling (102). The addition of *O*-GlcNAc to nuclear and cytosolic proteins is mediated by the *O*-GlcNAc transferase (OGT), whereas the enzyme *O*-GlcNAc-selective *N*-acetyl-beta-d-glucosaminidase (*O*-GlcNAcase) removes the *O*-GlcNAc, returning the protein to its basal state (103). *O*-GlcNAcylation has been shown to have extensive crosstalk with phosphorylation and to antagonize phosphorylation-mediated cell signaling (104).

In the pancreas, beta-cells are characterized by expressing high levels of the OGT enzyme. This allows these cells to dynamically respond to physiological increases in the extracellular glucose levels by converting glucose to UDP-GlcNAc, which is the OGT substrate, and therefore modulating intracellular *O*-linked protein glycosylation (105). In PDAC, hyper-*O*-GlcNAcylation has been associated with increased expression of the OGT enzyme and reduction of the *O-*Glc-NAcase glycosidase and has been demonstrated to block cancer cell apoptosis and to lead to the oncogenic activation of the NF-kB signaling pathway (106). Similarly, increased *O*-GlcNAcylation in colon has been demonstrated to contribute for the development of colitis and colitis-associated cancer by enhancing NF-kB-mediated signaling (107).

Along with aberrant protein glycosylation, cancer cells also display major glycosylation alterations on other classes of glycoconjugates, including the proteoglycans and the glycosphingolipids. Proteoglycans consist of a core protein with one or more covalently attached large GAG chains, and can be either located at the cell membrane or secreted. The syndecans are a family of transmembrane proteoglycans that carry heparan sulfate GAG chains and that can also be additionally modified with chondroitin sulfate chains (108). The heparan sulfate-rich proteoglycan syndecan-4, a critical partner of integrins for the establishment of focal adhesion complexes, has been shown to be upregulated in gastric mucosa in response to the oncogenic bacteria *Helicobacter pylori*. However, its functional role in the gastric carcinogenesis process remains to be disclosed (109, 110). Another syndecan family member, the syndecan-1, has been reported to be differently regulated and expressed in GI tumors. Loss of syndecan-1 expression has been described in gastric adenocarcinomas of higher stages (111), while in CRC and PDAC the expression of this proteoglycan has been shown to be upregulated, suggesting its possible application as a biomarker (112, 113).

Heparan sulfate GAG chains can also be carried by glypicans, a family of glycosylphosphatidylinositol (GPI)-anchored proteoglycans. Glypicans have been shown to bind a wide range of signaling molecules and to regulate the signaling of the Wnt, Hedgehog, fibroblast growth factor, and bone morphogenetic protein (BMP) pathways (114). Glypican-1 has been shown to be overexpressed in PDAC cell models and patient tumors (115). Moreover, the key role of glypican-1 in PDAC progression has been well documented using mouse models (116, 117). Recently, glypican-1 has been shown to be specifically expressed by cancer circulating exosomes and, therefore, to have potential to be used as a minimal-invasive diagnostic and screening tool to detect early PDAC stages (118).

The CD44 proteoglycan has also been on the focus of tumor biology research because the expression of specific splice variants is strongly associated with malignancy. Specifically, the exon v6-containing CD44 isoform (CD44v6) is highly expressed in premalignant and malignant gastric lesions (119). Modification of CD44v6 with STn was demonstrated in gastric mucosa and serum of cancer patients, indicating its potential as a biomarker for early diagnosis of gastric tumors (120). Different strategies aiming the impairment of CD44-dependent cancer cell migration have been proposed. The ceramide nanoliposome (CNL) was shown to induce anoikis and to limit metastasis by inducing lysosomal degradation of CD44 in PDAC cells (121).

Another glycosylation modification frequently observed in cancer is the altered sialylation of glycosphingolipids that can lead to the appearance of tumor-associated antigens. The human plasma membrane-associated sialidase NEU3, which catalyzes the removal of sialic acids from glycoproteins and glycolipids, is a key enzyme for ganglioside degradation. NEU3 has been shown to be overexpressed in many tumors, including CRC (122). Modulation of ganglioside expression by increased NEU3 activity has been proposed as a mechanism of protection against programed cell death and has, therefore, a critical implication in therapeutic strategies (123). Recently, NEU3 was demonstrated to regulate the Wnt signaling pathway, therefore contributing for the malignant transformation of CRC cells (124). These findings suggest NEU as a relevant target for diagnosis and therapy of CRC. During CRC progression, besides reduced expression of sialylated gangliosides, overall alteration in glycosphingolipids glycosylation includes increased fucosylation, decreased acetylation and sulfation, and reduced expression of globo-series glycans (125).

## Glycan Cancer Biomarkers

Glycosylation changes on glycoconjugates either expressed on the cell surface or secreted by cancer cells are potential sources of cancer biomarkers. The overexpression of these altered glycosylated structures and the loss of polarity of carcinoma cells lead to the shedding of glycoconjugates with altered glycosylation into the circulation. Currently, several serological assays used in the clinics are based on the quantification of glycoconjugate levels in the serum of cancer patients. Most of these biomarkers have been useful for prognostic and monitoring purposes. These include well-established serological biomarkers, such as the CA15-3 assay, detecting mucin MUC1 glycoprotein used for breast cancer (126–130), the CA125 assay, which detects the circulating mucin MUC16 in ovarian cancer (131, 132), and the prostate-specific antigen (PSA), which is used to detect prostate diseases (133, 134).

Regarding GI cancer, one of the most used serological assay detects the SLe^a^ carbohydrate antigen. SLe^a^ is present on circulating glycolipids and glycoproteins and is detected by the CA19-9 assay. This serological assay is applied in patients with a previously established diagnosis of PDAC, CRC, gastric, or biliary cancers and used to monitor their clinical response to therapy (135–138).

Another important serological test used in the clinics for GI tumors is the carcinoembryonic antigen (CEA) assay, which detects the CEA glycoprotein produced by carcinoma cells. In GI cancer, CEA is expressed at high levels and shed into the bloodstream being useful for prognosis evaluation and follow-up of these patients (129, 137, 139, 140).

In general, most of these serological assays have primarily been useful for prognosis and patients’ monitoring applications. Unfortunately, some of these biomarkers can also be detected due to benign lesions or other factors, such as smoking, which has limited their use in cancer screening strategies for diagnostic purposes. Given the usually late diagnosis of GI cancer, highly specific serum markers for cancer detection and screening are highly needed. Recent developed strategies and advanced technologies are contributing to the definition of novel and more specific glycoconjugate targets. Several of these new targets are currently evaluated and hold potential for improving the cancer detection and early diagnosis.

## Innovative Glycobiological Strategies

The difficulty of glycobiological research lies in the intrinsic complexity of glycosylation and its versatile conjugates. Whereas genomic and proteomic analysis made a leap forward by DNA sequencing and mass-spectrometric protein sequencing, respectively, that enabled the reading of a linear code with limited number of variabilities; for the more complex glycans, no comparable tool exists.

Nevertheless, the recent years have brought up many innovative approaches and methods that enable the unraveling of glycobiological challenges. With the development of glycoengineered cell strategies, glycan complexities have been reduced and the effects of specific glycan epitopes have been pinpointed. On the other hand, analytical methods and protocols for glycomic and glycoproteomic analyses have improved and new approaches, such as the adaptation of the array technology on glycans and lectins or novel antibody-based assays, have accelerated the acquisition of glycobiological knowledge. The following sections discuss several promising strategies in the glycobiology field.

### Glycoengineered Cell Line Models

The characterization of the function of glycans in cancer has been a major challenge in the field due to technical difficulties related to the complexity and heterogeneity of glycans synthesized in eukaryotic cells.

Genetic engineered cell models have been developed to study the functions of specific glycan epitopes in cancer. Some of these models include the overexpression of glycosyltransferases, which has allowed the characterization of the biosynthesis and function of simple cancer-associated carbohydrate epitopes, such as Tn, STn, T, and ST (17, 18, 20, 141, 142). Similar strategies have used stably transfected cell lines with glycosyltransferases to characterize the function of branched glycan structures (52, 56) as well as terminal sialylated/fucosylated structures frequently overexpressed by cancer cells, as previously explained in Section “Increased Sialylation and Fucosylation” (67, 143, 144).

Another major challenge in the field was related to the identification of structures at individual glycosylation sites. Major efforts have been done in this discipline with the generation of site-specific mutants of important proteins in cancer. One example is the use of site-specific mutants of *N*-glycosylation sites of the human epidermal growth factor receptor. This strategy has allowed the demonstration that Asn-420-linked oligosaccharide chain in this receptor interferes with its activation in cancer cell lines (145). Another cell line model has addressed the role of E-cadherin *N*-glycosylation sites in gastric cancer (146–148). The use of E-cadherin constructs engineered to lack specific *N*-glycosylation sites has demonstrated the effect of specific *N*-glycosylation structures on cell adhesion (149, 150).

The recent use of genomic editing tools has allowed the development of isogenic cell systems that along with extensive application of mass spectrometry (MS) methods is utilized for high-throughput site-specific *O*-Glycosylation (*O*-GalNAc and *O*-Mannose) proteomics. These technologies have enabled the precise determination of protein *O*-glycosylation sites in cells (151, 152). These strategies have greatly evolved in the past years and are showing vast potential in the glycobiology field. One approach has used the zinc-finger nucleases targeting the knockout of *COSMC* gene and has been applied in several human cancer cell lines originated from different organs (152). These so-called SimpleCell models produce stable cells expressing homogeneous truncated *O*-glycosylation with Tn and/or STn *O*-glycans (24, 120, 151, 153).

These cell models have provided a source of unlimited material for isolation and identification of GalNAc *O*-glycopeptides from cell lysates or secretomes using lectin chromatography followed by advanced MS, enabling the identification of hundreds of unique *O*-glycoproteins and *O*-glycosylation sites in several cell line models from different tissues (120, 153, 154). In addition, this approach has provided a versatile method for the functional analysis of different ppGalNAc-Ts (153, 155). Furthermore, similar strategies have been applied targeting the *O*-mannose glycoproteome. To reduce the structural heterogeneity of *O*-mannosylation (*O*-Man), the nuclease-mediated gene editing of a human cell line was performed by zinc-finger nuclease targeting of the *POMGNT1* gene. This gene encodes for the enzyme POMGnT1 that controls the first step in the elongation of *O*-Man glycans. The *O-*Man glycoproteome has been characterized using both chromatography and advanced MS (156).

The knowledge of *O*-glycosites in specific cancer cell types allows for the analysis of novel biological functions of glycosylation and for potential cancer cell-specific *O*-glycosites. This is particularly important given the complexity of *O*-glycosylation and that the various ppGalNAc-Ts that control the protein *O*-glycosylation sites may determine large variation at protein, cell, and tissue levels (9).

### Glycomic Strategy

Glycomics is the study of all glycan structures of a given cell, tissue, or organism. The intrinsic complexity of glycan structures and their versatile conjugates render this field particularly challenging. Due to the constant advancement of analytical instruments and methods, the *N*- and *O*-glycomic characterization of cancer cell lines, tumors, and cancer patients’ body fluids has rendered possible. Still, there is no single ideal method for this analysis and, thus, today a large variety of analytical methods is available for the glycomic characterization, resulting from different combinations of initial sample preparation, derivatization, glycan separation, and detection. Each method bares advantages and disadvantages.

**Figure 2:**
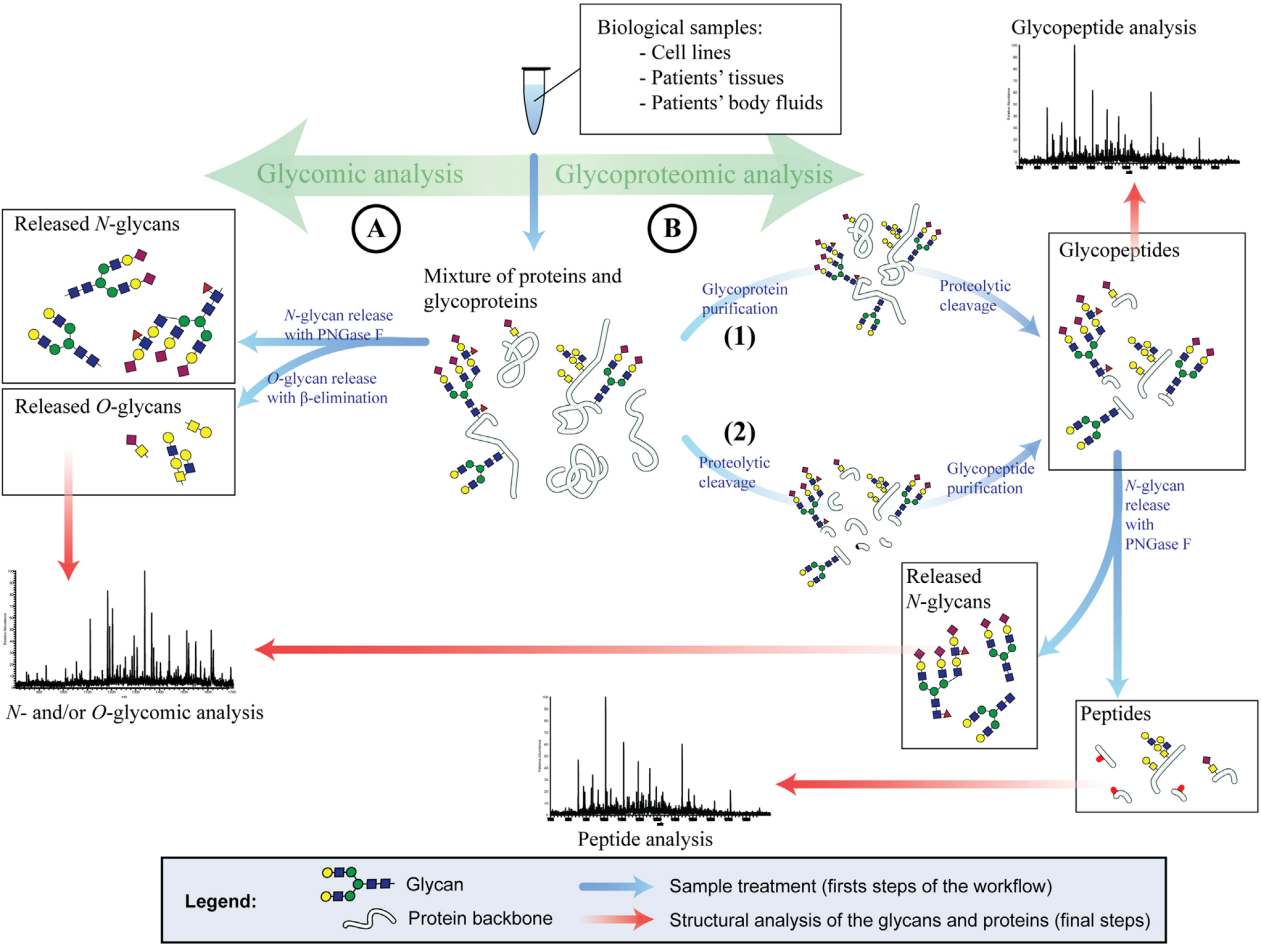
**Glycomic and glycoproteomic strategies for glycan analysis**. **(A)** Glycomic analysis. *N*- and *O*-glycans are PNGase F or chemically released, respectively. Once the glycans are released, they are structurally analyzed (usually by mass spectrometry). **(B)** Glycoproteomic analysis. The glycoproteins are either purified from a complex mixture and then enzymatically digested (1) or first digested and then purified (2). Method 1 will result in glycopeptides and non-glycosylated peptides originated solely from glycoproteins. Method 2 will result in glycopeptides only. These glycopeptides can be directly analyzed to generate site-specific glycan structures. Alternatively, the glycans of the glycopeptides can be released as described in **(A)** and the peptides are analyzed.

For the glycomic analysis of cells or tumors, the sample is usually homogenized and proteins are denatured, followed by the release of glycans (Figure 2A). The study of the glycans of serum or plasma is more challenging and requires often purification steps for glycoproteins prior to the release of their glycan structures. There are several methods to release glycans from the protein backbone to facilitate their characterization. The release of glycans is not a prerequisite as the analysis of whole glycopeptides is also possible (covered in Section “Glycoproteomic Strategy”). The most prominent technique to release *N*-glycans is by Peptide-*N*-Glycosidase F (PNGase F). The release of *N*-glycans via PNGase F is robust, fast, and efficient and is capable of liberating all types of human *N*-glycan structures. PNGase F-released glycans can be chemically labeled. On the other hand, no enzyme has so far been characterized that enables the efficient release of all types of *O*-glycans. For instance, the enzyme *O*-glycanase releases only core 1 *O*-glycans from their peptide backbone. Therefore, chemical techniques have to be utilized for whole *O*-glycomic analyses, such as reductive β-elimination.

Released glycans can be analyzed after derivatization or in their native form (underivatized). The derivatization of glycans bares several advantages, such as adding fluorescent tags for the photometric detection or chemical modification of side groups to stabilize glycan constituents. Despite these advantages, it is preferred in some cases to work with the native glycan, avoiding several time-consuming preparation steps and sample losses.

Complex mixtures of glycans, as they arise from clinical samples or even cell lines are usually separated by chromatography or capillary electrophoresis [reviewed in Ref. (157)] and detected by fluorescence detector (FLD) or MS. The sensitive and quantitative fluorescence detection requires fluorescently tagged glycans, and gives on its own only limited structural information derived from chromatographic or electrophoretic retention times. MS, on the other hand, can be applied on both native and derivatized oligosaccharides and may yield detailed structural information of the glycans. Since the analytes are not consumed by FLD, a sequential setup with MS is possible and often advantageous.

Three successful glycomic workflows that have revealed in the past years several findings in GI cancer are porous graphitized carbon separation with electrospray ionization and tandem MS (PGC-ESI-MS/MS), hydrophilic interaction ultra/high performance liquid chromatography with FLD (HILIC-FLD-UPLC/HPLC), and matrix-assisted laser desorption/ionization MS (MALDI-MS).

Porous graphitized carbon separation with electrospray ionization and tandem MS is a workflow used for both *N*- and *O*-glycomic analyses. First, the *N*-glycans are liberated from the glycoproteins with PNGase F, followed by reductive β-elimination of the glycoproteins to release the remaining O-glycans. The *N*- and *O*-glycans are separated by liquid chromatography with a PGC column, which resolves most isomeric structures and complements, therefore, ideally the subsequent MS and MS/MS structural analysis. A recent glycomic study by PGC-ESI-MS/MS has described structural glycan alterations in CRC, including several unique glycans found solely in the tumor region and indicated a correlation between EGFR expression and sialylation in CRC (158). This method has lately been further utilized for the *N*- and *O*-glycomic characterization of CRC cell lines and tumors, revealing great *O*-glycomic differences between tumors and all tested cell line models (26).

Hydrophilic interaction ultra/high performance liquid chromatography with FLD is used for the quantitative profiling of *N*-glycans. The *N*-glycans are released by PNGase F and reductively aminated with a fluorophore. The labeled *N*-glycans are applied on a HILIC-ultra performance chromatography (UPLC), which separates the glycans according to their size and monosaccharide composition. The retention time can be converted to glucose units (GU) by comparing it with a dextran ladder, yielding reproducible results. Hence, due to the few sample preparation steps, the high recovery of the HILIC column, the quantitative detection via the fluorescent tag, and the possibility of multiplexing, this analysis can be applied for large-scale *N*-glycomic studies. The HILIC-FLD-UPLC *N*-glycomic analysis has recently been applied in a large-scale discovery study on serum of gastric cancer patients revealing an increase in certain SLe^x^ carrying *N*-glycan structures that correlated with disease progression. Furthermore, in this study other structures, such as bisected *N*-glycans, have been shown to decrease with disease progression (159, 160).

Recently, the combination of HILIC-FLD-UPLC and PGC-ESI-MS/MS has been used for *N*- and *O*-glycomic analysis of a gastric cancer cell line overexpressing the sialyltransferase ST3Gal-IV (79). This cell line has previously been shown to present a more invasive phenotype (67). The glycomic analysis revealed a broad range of cancer-associated alterations, such as decreased bisected and increased branched structures, truncation of *O*-glycans, and a shift from α2,6- to α2,3-sialylated *N*-glycans (79).

The use of MALDI-MS is another very successful approach of analyzing the *N*-glycome of clinical samples, such as body fluids. MALDI is based on a laser impulse that excites a solid matrix in which the analytes are embedded which in turn desorbs and ionizes the analytes for MS analysis. MALDI is relatively tolerant to salt and other contaminants, which allows uncomplicated sample preparation after the release of *N*-glycans. This method has recently been applied on the serum of gastric cancer patients and control groups and has been able to identify *N*-glycomic differences between the serum of gastric cancer patients and that of non-atrophic gastritis patients (161). In a large-scale study on sera of PDAC patients, a tendency toward higher branched and fucosylated *N*-glycans has been observed when compared to sera from healthy individuals. The major part of the significantly altered *N*-glycan structures were specifically increased in patients with distant metastases and the ratio of the quantity of two glycans has been proposed as a robust diagnostic marker for PDAC (162). Another recent study utilizing MALDI-MS has revealed in pancreatic cyst fluids, of which certain subtypes bare a high risk of undergoing malignant transformation, the hyperfucosylation of *N*-glycans (163).

A broad range of alterations in CRC tissues versus controls have been identified by sequential analyses of fluorescently tagged *N*-glycans by HILIC-FLD-HPLC and MALDI-MS. Additionally, multivariate statistical evaluation and further MS-based structure elucidation have been applied and revealed among others the decrease of bisected structures and the increase of glycans with sialylated lewis epitopes. Furthermore, abnormal core-fucosylated high mannose *N*-glycans have been uniquely found in cancer tissue (37).

### Glycoproteomic Strategy

Glycoproteomics is the study of proteins that carry glycan modifications. It usually focuses on the identification and quantification of glycoproteins and the characterization of protein glycosylation sites. Given that most clinical cancer biomarkers are glycoproteins, this field is particularly promising for the identification of new biomarker targets in cancer. Biological samples, such as cell lines, tissues, and body fluids, can be analyzed. However, the glycoproteomic analysis of complex biological samples, such as tissues or sera, is analytically challenging due to the large complexity and vast dynamic range of concentrations of glycoproteins.

The glycoproteomic pipeline typically consists of numerous steps, such as glycoprotein or glycopeptide enrichment, isotopic labeling (optional), multidimensional protein or peptide separation, tandem mass-spectrometric analysis, and bioinformatic data interpretation (Figure 2B). In cancer, the vast majority of glycoproteomic findings are based on bottom-up analysis of peptides (“shotgun proteomics”). For this purpose, glycoproteins are proteolytically cleaved into glycopeptides before or after the enrichment step. The enrichment of glycoproteins or glycopeptides is a critical step of the glycoproteomic analysis. Even though this field is rapidly evolving, so far no method has been established that captures unbiased every glycoprotein or glycopeptide and enables full glycoproteomic coverage. Currently, most popular enrichment methods are based on lectins (164–166) or on hydrazide solid-phase extraction (167, 168) and sometimes applied in combination to increase the glycoproteomic coverage (168). Alternative strategies are boronic acid functionalized beads (169), size exclusion chromatography (170), hydrophilic interaction (171), and graphite powder micro column (172). Due to the difficulties of covering the whole glycoproteome many cancer studies pursue a different strategy of enriching specifically glycoproteins and glycopeptides carrying cancer-relevant glycan epitopes, such as sialic acids or sialylated Lewis epitopes. These methods are usually based on lectins [such as SNA, WGA, and MAL (173)], antibodies (159), enrichment by titanium dioxide (79, 174), or affinity purification of metabolic labeled glycoproteins (175–177). After the enrichment and proteolytic digestion (not necessarily in this order), glycopeptides may be deglycosylated and are multidimensional separated via chromatography and/or electrophoresis and analyzed by tandem MS. The deglycosylation is a requirement of some enrichment methods, such as hydrazide solid-phase extraction, but may be also applied for all *N*-glycoproteomic analysis. The PNGase F release of *N*-glycans leads to the conversion of the *N*-glycan carrying asparagine to aspartic acid and can, thus, be spotted on the peptide backbone by MS. For the generation of site-specific structural information of *N*- and *O*-glycans, whole glycopeptides are analyzed utilizing a combination of different MS fragmentation methods or collision energies that either fragment peptides or glycans (178–180). This strategy is being optimized in recent years and bares great potential for the discovery of new cancer biomarkers because it unravels site-specific glycan alterations in cancer.

Glycoproteomic analyses have been applied in GI cancer mainly for the identification of biomarkers, such diagnostic biomarkers or biomarkers for multidrug resistance in gastric cancer (181, 182). Glycoproteomics in combination with glycoengineered cell line models was in recent years able to increase the coverage of *O*-glycosylated proteins and to identify numerous novel *O*-glycosylation sites in gastric cancer and PDAC, generating several new potential biomarkers (24, 120).

### Other Glycoanalytical Techniques

MS-based glycomic and glycoproteomic analyses require expensive equipments and a fair amount of expertise. MS-independent methods, such as glycoprotein, antibody-lectin-sandwich, and lectin arrays, are capable of rapid data acquisition of glycomic alterations in cancer samples. Glycan arrays, on the other hand, enable a screening for specificities of glycan-binding proteins, improving the data interpretation of antibody and lectin-based research. Regarding tumor biology, it is very relevant to determine not only the glycosylation modifications harbored by tumor cells but also to disclose the topographic distribution of these alterations within the tumor and adjacent tissue. Novel approaches for the identification of *in situ* glycan modification of specific proteins include proximity ligation assay (PLA) and imaging mass spectometry (IMS).

#### Arrays

**Figure 3:**
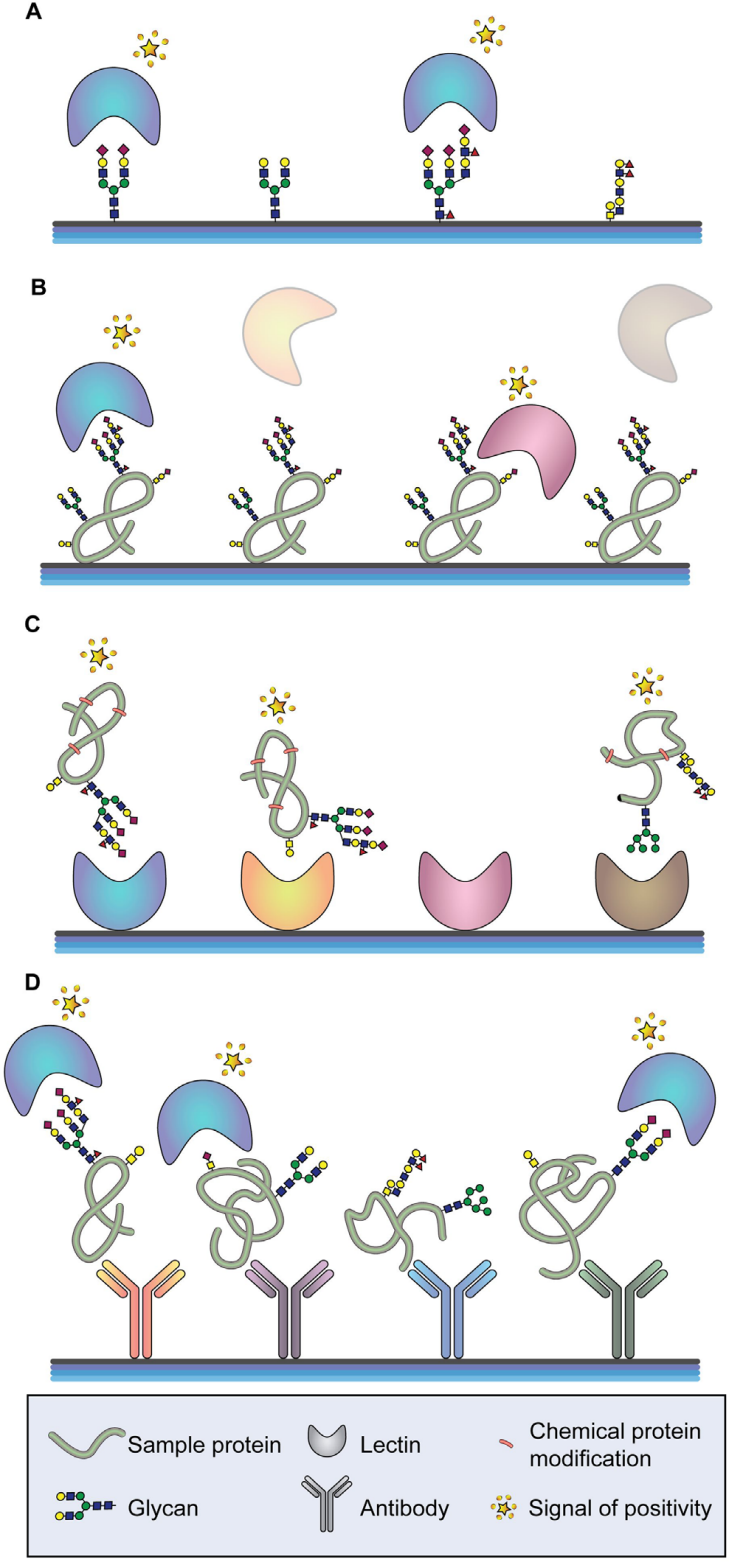
**Arrays for glycan analysis**. **(A)** Glycan arrays. Different glycan moieties are spotted onto the array to determine specificities of labeled glycan-binding molecules (typically lectins or antibodies). **(B)** Glycoprotein arrays. Glycoproteins are enriched from a sample and spotted onto the array. The glycosylation moieties of the spotted glycoproteins are determined by screening with different glycan-binding molecules. **(C)** Lectin arrays. A series of lectins with well-defined glycan-binding properties are spotted onto the array and different labeled proteins are tested. **(D)** Antibody-lectin sandwich arrays. Multiple antibodies for a series of proteins of interest are spotted onto the array, and the glycan epitopes of the captured proteins are probed using labeled lectins and glycan-binding antibodies.

The binding of biological molecules to solid matrixes was an idea first described by Chang in 1983 (183). This technology initially consisted of coating glass cover slips with different antibodies in close proximity forming a matrix-like array. Arrays recognize partners from large amounts of biological material using high-throughput screening miniaturized, multiplexed and parallel processing, and detection methods based on multiple probes covalently attached to a solid substrate. Depending on the molecule that is deposited on the surface, different microarrays exist. To analyze glycan-containing structures, the most common classification is glycan, glycoprotein, or lectin microarray, and also a variant of the latter called antibody-lectin sandwich array (Figure 3) (184). The advantages that the microarray technology offers are the small volume of sample required for the analyses, the high reproducibility, and the reduced cost and time to process many samples. Therefore, microarray platforms have been highlighted by its extensive application in the field of biomarker validation, where a large number of samples must be analyzed multiple times (185). Moreover, depending on the type of microarray assay performed, information about the glycan-linkage configuration can be obtained.

Glycan microarrays are used mainly to characterize the binding specificities and affinities of proteins (mostly antibodies and lectins) toward glycans (Figure 3A) (186–188). However, they can also be applied for the screening of inhibitors of carbohydrate-mediated interactions and of sugar interactions of an entire organism, such as a whole cell or virus (185, 189, 190). Current available platforms consist of approximately 20,000 microspots of antigens reaching the capacity to include most known human microbial pathogens, autoantigens, and tumor-associated antigens (191–193). The diversity and scope of glycan arrays are continuously increasing allowing a better characterization of glycan-binding proteins but leading to more complex data. Different software tools are currently available for data interpretation (194–196). Glycan arrays present oligosaccharides that were either purified from a biological source or *de novo* synthesized. Regarding the latest ones, it is important to highlight recent works describing new methodologies that allow sialic acid (197, 198) and GAG synthesis (199).

As an alternative to the direct binding of glycans to the array surface, glycans can be presented on proteins or peptides that are attached to the array. A recent advancement in this approach is the coiled coil-based technology, which allows the presentation of the antigens at high densities while mimicking the *in vivo* orientation attached to a fiber-forming peptide. This platform showed increased sensitivity for the identification of antibodies against parasitic glycan antigens and might be adapted in the future for cancer diagnostic (200).

Glycoprotein microarrays are based on printing purified or enriched glycoproteins onto the slides and screening these proteins for glycan epitopes using different lectins or glycan-recognizing antibodies (Figure 3B). This approach is usually followed by analytical techniques to identify the spotted proteins and to verify the glycan epitopes found by the array analysis. A recently performed glycoprotein array analysis of lectin-enriched sera from PDAC patients, chronic pancreatitis patients (benign pancreatic disease), and healthy individuals has correctly clustered these three groups, being the PDAC group significantly different from the other two (201). In addition, the glycoprotein microarray may use synthesized peptides and recombinant protein fragments that have been *in vitro* glycosylated for the detection of human autoantibodies (202, 203).

Lectin microarrays, where different lectins are spotted onto the slide, enable a rapid and high-sensitivity profiling of glycan features found in complex samples, such as cells, tissues, body fluids, and synthetic glycans and their mimics (Figure 3C) (204, 205). Lectin arrays offer a general view of the glycan structures on a complex sample and integrate the information from all proteins with the disadvantage that no information about specific glycan changes of the respective protein constituents will be obtained (206). A recent work displayed different glycopatterns in gastric cancer compared to gastric ulcer applying Cy3-labeled proteins extracted from tissues to lectin microarrays (207). Another recent lectin array approach has identified differences in α2-macroglobulin glycosylation between healthy individuals and patients with CRC. The spotted serum purified α2-macroglobulin has displayed, among other changes, significant differences in the content of branched *N*-glycans and α2,6 sialylation (208).

A variant of the lectin array is the antibody-lectin sandwich array. Antibodies to known glycoproteins are spotted on a solid support, and complex glycoprotein samples, which can be crude or prefractionated, are bound to the microarray (Figure 3D) (184, 185). The glycosylation of the captured target proteins are then screened by labeled lectins and glycan-specific antibodies. Antibody-lectin sandwich arrays are highly effective for profiling variation in specific glycans on multiple target proteins. Performing this technology, specific glycoforms of MUC5AC and endorepellin glycoproteins in the cyst fluid of patients with precancerous pancreatic cysts have been found (209). In these assays, the glycoprotein nature of the antibodies must be considered and different approaches to prevent glycan recognition of the antibodies by the secondary antibody or lectin applied exist. The chemical derivatization of the glycans of the spotted antibodies also prevents their ability to be recognized by glycan-binding molecules, both antibodies and lectins. One efficient method to study glycans on individual proteins from complex mixtures uses chemically derivatized capture antibodies and tests the glycosylation of captured target proteins by lectins and glycan-binding antibodies. Applying this approach, cancer-associated glycan alterations on the proteins MUC1 and CEA in the serum of PDAC patients have been identified (210). Another strategy consists on producing recombinant antibodies in organisms that do not carry out post-translational modification, such as glycosylation. This approach has been performed for the detection of glycans linked to CEA by ELISA coating the microplate with recombinantly scFv expressed in *Escherichia coli* and using lectins as detection probes (211).

Regarding GI cancer research, arrays have been widely used to discover new biomarkers consisting of proteins bearing aberrant glycosylation that could lead to a more accurate diagnostic.

#### *In situ* Proximity Ligation Assay

**Figure 4:**
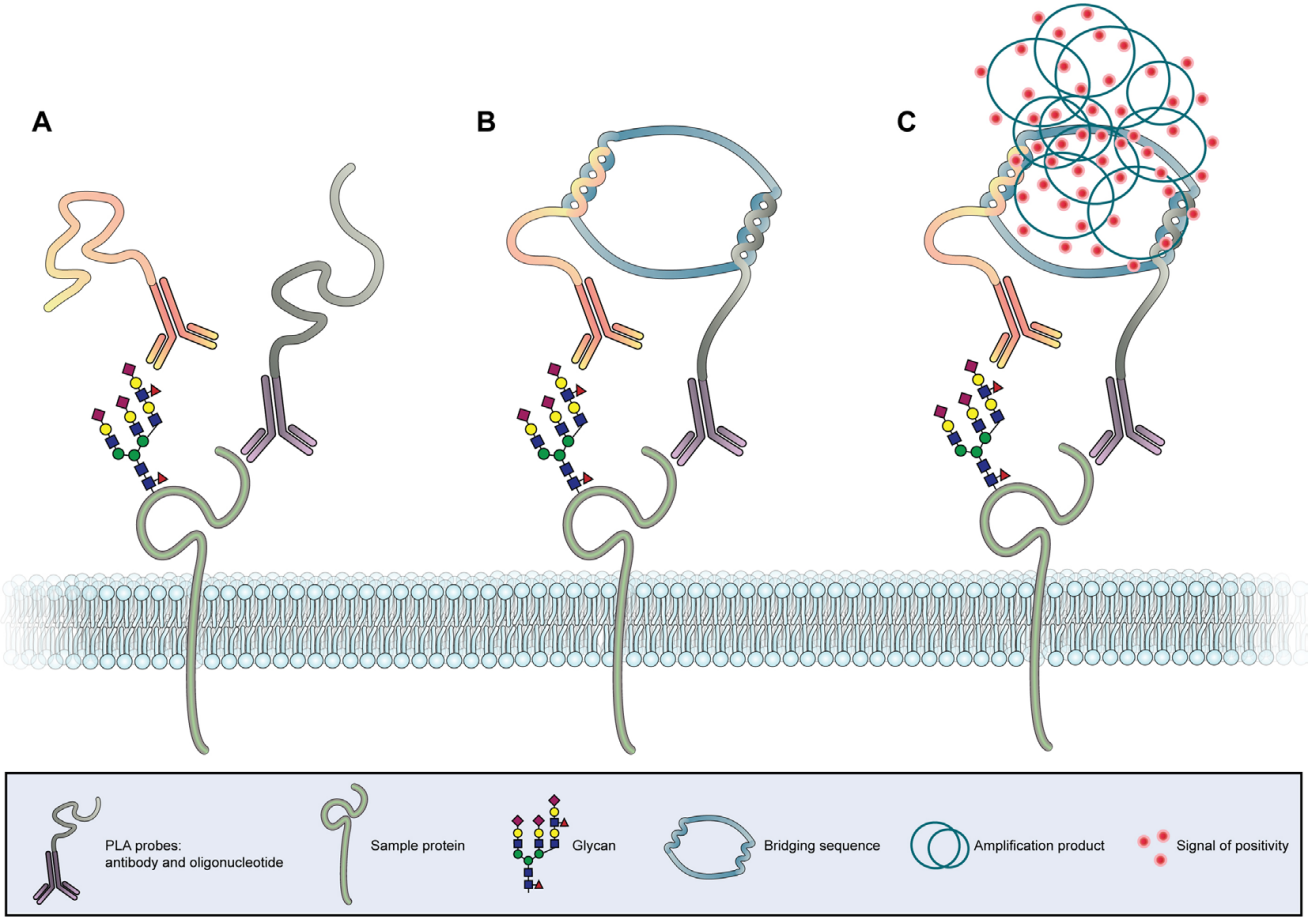
***In situ* proximity ligation assay**. **(A)** Two antibodies, one specific for the protein backbone and another specific for a glycan epitope, are conjugated with two different oligonucleotide chains (PLA probes). **(B)** In case the antibodies bind to molecules in close proximity a bridging sequence links the two oligonucleotide sequences. **(C)** A subsequent polymerase induced amplification in combination with labeled nucleotides leads to the formation of a fluorescent or chromogenic signal at the co-expression site of the protein and glycan, allowing the *in situ* detection of the PLA signal.

The association of the glycan expression and location with clinical and molecular characteristics of cancer tissues has rendered possible by histochemistry techniques using glycan-binding antibodies or lectins (205). However, one major limitation of this technique is the lack of capacity to identify the proteins *in situ* on which these glycan motifs are localized. This limitation has been surpassed by the development of the *in situ* proximity ligation assay (PLA) (Figure 4) (212, 213).

This sensitive antibody-based method reveals the colocalization between specific proteins and specific glycan structures in tissues and cell samples. The identification of protein glycoforms is of utmost importance for the understanding of glycobiological cellular processes in cancer. PLA could also detect other post-translational modifications of proteins in tissue samples with subcellular resolution. The PLA technology is based on the binding of two specific PLA probes, each containing a unique oligonucleotide, to two targets of interest (Figure 4A). Antibodies, lectins, and other binding proteins can act as probes. A ligation solution, containing bridging oligonucleotides and a ligase, will hybridize the oligonucleotides of the PLA probes if they are in close molecular proximity to form a closed circle (Figure 4B). This closed nucleotide circle will be amplified by a DNA polymerase generating repeated copies of the circular DNA strands. Finally, fluorescent or chromogenic oligonucleotides hybridize to the amplification product and can be detected as individual spot by microscopy (Figure 4C) (213). The first study using this innovative PLA strategy applied to glycobiology has showed that the mucin MUC2 is a major carrier of the cancer-associated STn glycan antigen both in intestinal metaplasia and gastric carcinoma (214). The use of *in situ* PLA for the identification of a mucin glycosylation profile in cancer lesions is being extended, opening new opportunities for the development of novel diagnostic and prognostic markers. One recent study screened for tissue-specific aberrant mucin glycoforms in mucinous adenocarcinomas from different organs (stomach, ampulla of Vater, colon, lung, breast, and ovary). In GI tissues mucins carrying a set of truncated, simple O-glycans and sialylated Lewis antigens have been detected by this approach (215).

More recently, PLA has been used in combination with different glycoproteomics strategies to identify specific glycoforms as potential biomarkers in gastric cancer, leading to the identification of CD44v6/STn (120) and RON/SLe^x^ (79).

The PLA technique will further improve our understanding of specific protein glycosylation changes that occurs in cancer tissues and that could be applied in clinic as new markers for GI cancer progression (216).

#### Imaging Mass Spectrometry

**Figure 5:**
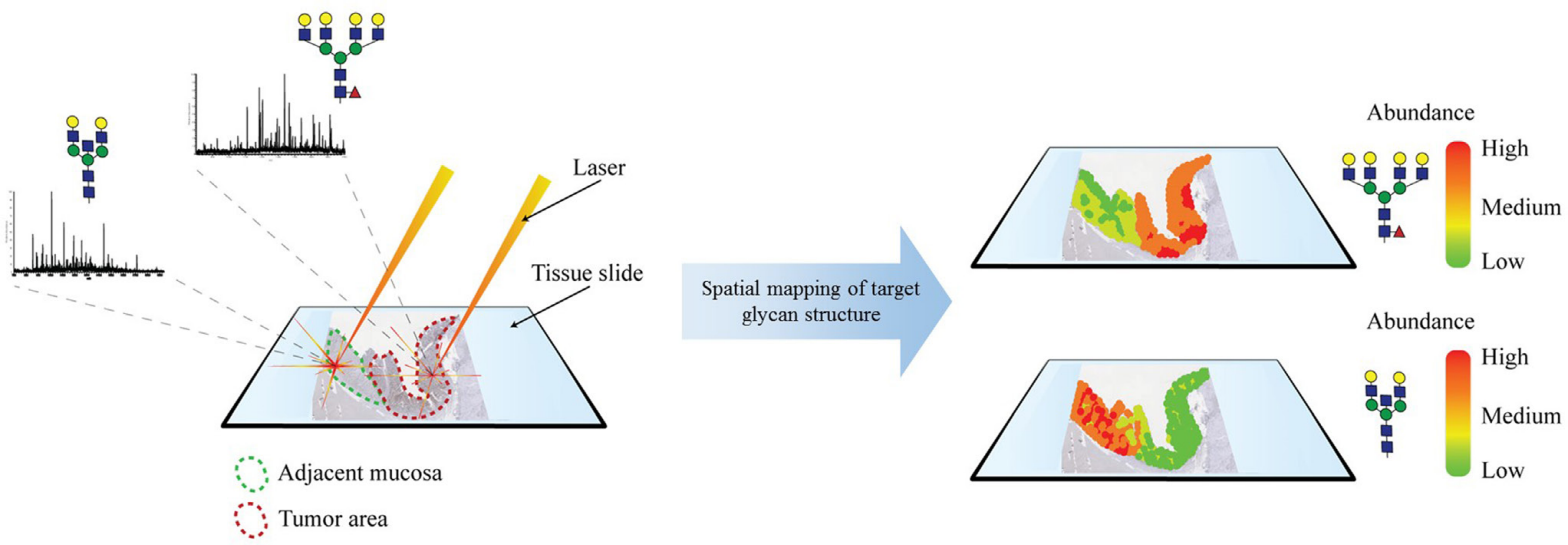
**Imaging mass spectrometry**. MALDI-based ionization generates an MS glycan spectrum of a selected spot of a tissue section. This process is repeated throughout the tissue. Identified glycan structures are mapped on the tissue and can be correlated with histopathological features.

Imaging mass spectrometry is a very novel and promising technology that was first developed in 1997 by Caprioli and colleagues for the analysis of proteins (217). This method is based on MALDI-MS and utilizes the laser ionization of a localized area for the two-dimensional screening of a tissue sample. IMS generates for each ionization point of the tissue a spectra that yields structural information and, thus, reveals the spatial distribution of analytes (Figure 5). Recently, this technique has been adapted for glycomic analysis and has allowed to create *N*-glycosylation maps of several different frozen tissue (218). Following, *N*-glycan IMS has been also applied on formalin-fixed paraffin-embedded tissue (219). The *N*-glycan IMS workflow consists of four steps. First, *N*-glycans are liberated by PNGase F incubation of the deparaffinized or thawed tissue slide. Second, a thin layer of MALDI matrix is sprayed on top of the tissue slide. Third, the slide is two-dimensionally screened by multiple MALDI-MS analysis. Lastly, each identified *N*-glycan structure can be computationally visualized on the tissue, generating an epitope map.

IMS applied on hepatocellular carcinomas has shown to be capable of spatially defining glycan compositions and distinguishing malignant tissue from healthy tissue (220). Preliminary IMS results on other tumors, such as PDAC, have been able to differentiate between histopathological areas, such as fibroconnective tissue (220).

The IMS application on *N*-glycan analysis is still in its early stages of development, but bares enormous potential as a next-generation *N*-glycomic tumor characterization tool.

## Future Perspectives and Clinical Applications

The recent advances in the glycomic and glycoproteomic fields are currently providing crucial information on the understanding of the role that glycans play in the biology of cells, tissues, and organisms, both in physiological and pathological conditions. However, many issues still remain to be understood, particularly in complex diseases, such as cancer. Advances in the glycobiology field could contribute to disclose key information regarding cancer biological properties, including the identification of prognostic and therapeutic response biomarkers.

In addition, the recent developments in this field could contribute to overcome the limitations of the current serological assays. The set of novel strategies presented in this review provide a clear view for future validation of potential biomarkers and points toward the translation of these strategies in the clinical setting.

## Author Contributions

All authors have contributed to the conception, drafting, and revision of the manuscript. SM and MB designed the figures. All authors approved the review final form.

## Conflict of Interest Statement

The authors declare that the research was conducted in the absence of any commercial or financial relationships that could be construed as a potential conflict of interest.
